# Insights into the biocompatibility of biodegradable metallic molybdenum for cardiovascular applications-a critical review

**DOI:** 10.3389/fbioe.2024.1457553

**Published:** 2024-09-23

**Authors:** Janina Mayers, Brianna Hofman, Indie Sobiech, Maria P. Kwesiga

**Affiliations:** ^1^ Department of Biomedical Sciences, Grand Valley State University, Allendale, MI, United States; ^2^ Department of Cell and Molecular Biology, Grand Valley State University, Allendale, MI, United States

**Keywords:** atherosclerosis, biocompatibility, biodegradable metal stents, molybdenum, xanthine oxidoreductase

## Abstract

Atherosclerotic cardiovascular disease (ACD) is the leading cause of death worldwide. The gold standard of treatment is the implantation of a permanent stent implant that is often associated with complications such as thrombus formation, vascular neointimal response, and stent fracture, which altogether decrease the long-term safety and efficacy of the stent. Biodegradable metallic materials have become an attractive alternative because of the ability to facilitate a more physiological healing response while the metal degrades. Recently, Molybdenum (Mo) has been considered as a potential candidate due to its excellent mechanical and medical imaging properties. Moreover, the biomedical research studies performed to date have shown minimal adverse effects *in vitro* and *in vivo*. However, there are still concerns of toxicity at high doses, and the impact of the biochemical mechanisms of Mo on material performance especially in pathophysiological environments are yet to be explored. Mo is an essential co factor for enzymes such as xanthine oxidoreductase (XOR) that plays a critical role in vascular homeostasis and ACD progression. Herein, this review will focus on the biochemistry of Mo, its physiological and pathological effects with an emphasis on cardiovascular disease as well as the recent studies on Mo for cardiovascular applications and its advantages over other biodegradable metals. The limitations of Mo research studies will also be discussed and concluded with an outlook to move this revolutionary metallic biomaterial from the bench to the bedside.

## 1 Introduction

Atherosclerotic cardiovascular disease (ACD) is the leading cause of death worldwide and has been recognized as a significant threat to development in the 21st century (Khan et al., 2020). ACD is characterized as the buildup of plaque in arterial walls which consists of lipids, fragments of dead cells, calcium salts and other fatty molecules (Rafieian-Kopaei et al., 2014). The first line of non-surgical treatment for patients with advanced and acute manifestations of ACD is percutaneous coronary intervention (PCI) with deployment of a permanent stent (Khan and Ludman, 2022). Stents were designed to offer structural support while restricting sudden contractions and vessel blockage (Wright et al., 1985; Jeewandara et al., 2014). However, since the stent persists in the body, it provokes a chronic inflammatory response (Jukema et al., 2012). More recently, biodegradable materials have been proposed as an alternative to permanent stent implants (Borhani et al., 2018). Ideally, a biodegradable stent would have the following properties: minimal adverse effects when in contact with cells of the body, the ability to withstand compression and tensile forces, an optimal degradation rate which does not trigger a hostile inflammatory response, and the ability to remain functional with a relatively small strut size (Borhani et al., 2018; Wayangankar and Ellis, 2015). The current biodegradable metals under investigation include Magnesium (Mg), Iron (Fe) and Zinc (Zn). Mg is at the forefront and has recently been assessed in clinical trials (Haude et al., 2016; Sabaté et al., 2019; Verheye et al., 2021; Ghafari et al., 2023) Although, the accelerated degradation rate has led to its underperformance (Yang et al., 2018; Ng and Loh, 2019; Bossard et al., 2022). Molybdenum (Mo) shows promise as a biodegradable metal stent since it independently presents with excellent mechanical properties, uniform degradation rate, and medical imaging capabilities (Redlich et al., 2021; Schauer et al., 2021; Sikora-Jasinska et al., 2022). There are concerns of Mo toxicity especially at high doses. However, the cardiovascular research studies performed to date have shown minimal toxic effects *in vitro* and *in vivo.*

Mo is a bio essential element which is a co factor of enzymes that are necessary to maintain various homeostatic functions in the body. One of these enzymes’ xanthine oxidase reductase (XOR) is found in cardiac, vascular endothelial cells as well as macrophages and it is a key mediator in generating oxidative stress during ACD (He et al., 2019; Polito et al., 2021). The development of ACD mainly involves endothelial cells, macrophages, and vascular smooth muscle cells in the intimal layer of the blood vessels. The oxidation of low-density lipoprotein cholesterol (Ox-LDL) initiates the production of chemokines and expression of adhesion molecules on endothelial cells, which make the intima more permeable to Ox-LDL and circulating monocyte migration which differentiate into macrophages (Kume et al., 1992). The activated macrophages ingest Ox-LDL and become foam cells leading to atheroma formation (Chistiakov et al., 2017). Foam cells produce growth factors that cause the vascular smooth muscle cells to proliferate and migrate into the vessel lumen forming a partial blockage (stenosis) that interferes with blood flow (Alonso-Herranz et al., 2023). The persistent inflammatory response elicited by permanent stents consequently increases the risk of in-stent restenosis (ISR) (Jukema et al., 2012; Borhani et al., 2018). The hope is that a biodegradable Mo stent would gradually restore vascular function and theoretically reduce the risk of ISR since it will degrade appropriately over time.

The limitations of previous studies are that they do not account for the biological effects of Mo that could significantly impact the material and host interaction required for the success of stent implants especially in the presence of ACD. Research that has been done on Mo attributes its toxicity in part to over activity of these Mo containing enzymes (Vyskočil and Viau, 1999). In addition, there is cross talk that exists between Mo and other metallic elements such as copper (Cu) (Schwarz et al., 2009) which would potentially alter Cu metabolism and its biological effects downstream. These biochemical mechanisms of Mo are underrepresented in current biomedical research and could potentially have implications at the molecular level that could hinder the expected performance of Mo biomaterials. In this review, we focus on the biochemistry of Mo, its physiological and pathological effects, the recent studies of Mo for cardiovascular applications, comparison with other biodegradable metals, and areas that need to be explored.

## 2 The biochemistry of molybdenum

**FIGURE 1:**
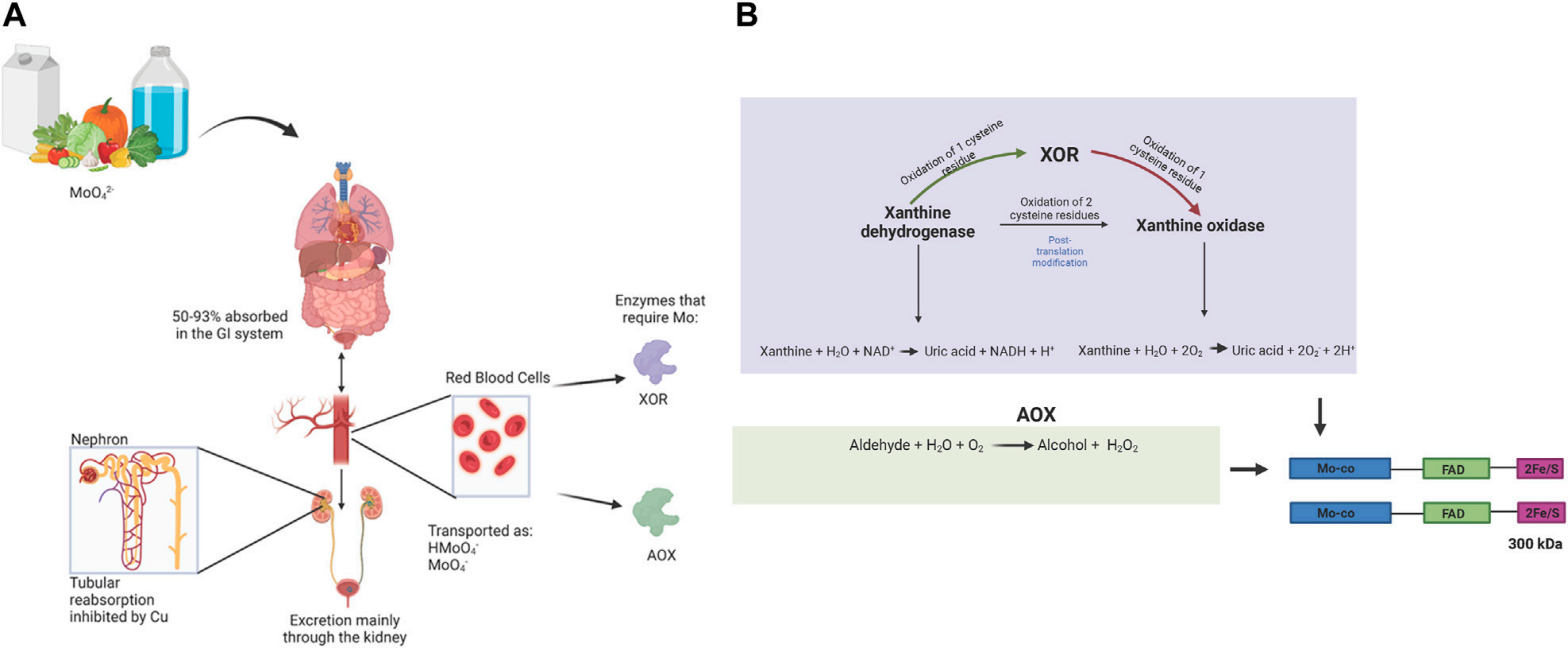
**(A)**: Mo Bioavailability: Mo containing dietary products (vegetables, milk and drinking water) are absorbed within the gastrointestinal tract and incorporated into blood circulation (Barceloux, 1999). Transport within erythrocytes is via Cl^−^/HCO_3_ anion exchanger with Mo in the form HMoO_4_^−^/MoO_4_^2-^ (Gimenez et al., 1993). In different organs and tissues, Mo is incorporated into the Mo-co subunit of the Mo containing enzymes (molybdo-flavoenzymes); xanthineoxidoreductase (XOR) and aldehyde oxidase (AOX). Mo is mainly secreted in the kidneys through an antagonistic mechanism of Cu and the Mo protein transporter (Barceloux, 1999). **(B)**: Biochemistry XOR and AOX: Xa nthine oxidoreductase (XOR) is a derivative of the enzyme xanthine dehydrogenase (Polito et al., 2021). XDH undergoes post translational modification through oxidation of cysteine residues, which depending on the number of groups oxidized, can either be converted into XOR (oxidation of 1) which expresses both dehydrogenase and oxidase activity or XO (oxidation of 2) (Bortolotti et al., 2021). The enzyme XOR exists as a homodimer that consists of 2 identical subunits with 3 domains, a Mo-co (85 kDa) which acts as the electron donor, an FAD (45 kDa) which acts as the electron acceptor and 2Fe/S (20 kDa) which mediates the transfer of electrons from Mo-co to FAD (Bortolotti et al., 2021). The different isoforms of this enzyme are all involved in the last two steps of the purine metabolism pathway that converts xanthine into uric acid (Polito et al., 2021; Bortolotti et al., 2021). The difference lies in the cofactors that mediate this reaction, XDH uses NAD+ and XO has a greater affinity for oxygen. Aldehyde oxidase shares 86%–91% gene sequence homology with XOR and consequently has a similar protein structure (Kelley, 2017). The AOX enzyme oxidizes aldehyde compounds into alcohol and it also oxidizes aromatic compounds simultaneously generating hydrogen peroxide (H_2_O_2_) (Terao et al., 2016; Terao et al., 2020).

Molybdenum (Mo) is a transitional metal element that was first discovered in 1778 (Barceloux, 1999). Mo naturally exists in 5 oxidation states Mo (-II)-Mo (+VI) and can also exist in the metallic form (Barceloux, 1999; Saji and Lee, 2012; Burzlaff et al., 2017). In dilute solutions it exists mainly as soluble MoO_4_^−2^ (Barceloux, 1999). With increasing concentrations, Mo will polymerize into polymolybdate complexes (Barceloux, 1999). Mo has been classified as an essential element (Bibr et al., 1960; Oskarsson and Kippler, 2023). It is abundantly found in leafy vegetables, legumes, cereal, cauliflower, kidney, liver, and milk (Barceloux, 1999; Bibr et al., 1960). Approximately 50%–93% of soluble Mo is absorbed in the gastrointestinal tract and transported in the blood to the kidney and the liver (Barceloux, 1999). Body transport occurs through erythrocyte cells particularly via a Cl-/HCO_3_ anion exchanger in form of HMoO_4_^−^ and to a lesser extent, MoO_4_^2-^ (Gimenez et al., 1993). The uptake of Mo in cells is through Mo transporters (MOT1 and MOT2). MOT1 is exclusively found in plants, specifically in the endoplasmic reticulum while MOT 2 has been identified in animals and humans within intracellular vacuoles (Tejada-Jiménez et al., 2011; Mendel and Kruse, 2012). The majority of Mo is then excreted via the kidneys (Vyskočil and Viau, 1999). Studies have also shown that Mo is excreted in bile and feces in small proportions (<1%) (Barceloux, 1999; Vyskočil and Viau, 1999). High Cu levels facilitate the excretion of Mo by inhibiting the reuptake of Mo through its transport protein (Barceloux, 1999) (Figure 1A) Mo has been shown to bind to the erythrocyte membrane protein spectrin (Kselíková et al., 1980) and it has also been reported to bind strongly to the serum protein alpha-2-macroglobulin, an acute phase reactant which plays a role in mediating phagocytosis in macrophages during inflammation (Vandooren and Itoh, 2021).

The diversity in oxidation states of Mo makes it a critical element in catalyzing redox reactions in cells. Mo is a well-known cofactor for several enzymes (Maia et al., 2024). The cofactors can be classified into two: 1) Iron–sulphur-cluster-based iron–Mo cofactor (FeMo-co) found in Mo nitrogenase enzymes which break down dinitrogen (N_2_) into ammonia (NH_3_) and 2) a pterin-based cofactor, molybdopterin (Mo-co) and is found in sulphite oxidase (SO), xanthine oxidase (XO) and dimethylsulphoxide reductase (DMSOR) (Schwarz et al., 2009). In eukaryote organisms, the predominant form is the pterin-based co factor Mo-co. This review will discuss the XO family which has implications in ACD pathophysiology. (Patetsios et al., 2001; Berry and Hare, 2004; Nomura et al., 2014; Polito et al., 2021) Information regarding the biochemistry of Mo nitrogenase, SO, and DMSOR can be found elsewhere (Schwarz et al., 2009).

The XO family, also known as Mo hydroxylases is a family of enzymes that consist of a homodimer structure that contains two 150 kDa subunits, each with 3 specific binding domains: flavine adenine nucleotide (FAD), 2 iron/sulphur cluster group and Mo-co (Schwarz et al., 2009). There are two major classes of enzymes in this family. Xanthine oxidoreductase (XOR), which can also exist in two interconvertible isoforms (Figure 1) xanthine dehydrogenase (XDH) and xanthine oxidase (XO) (Harrison, 2002). The enzyme form xanthine dehydrogenase (XDH) is the native form and is ubiquitous to most living organisms (Battelli et al., 2018). The XO isoform only exists in mammals, under conditions such as hypoxia or inflammation (Harrison, 2002; Kelley, 2017). Here onwards, the enzyme will be referred to as XOR in mammalian studies. The second class are the aldehyde oxidases (AOX). The major difference between the 2 classes of enzymes is substrate specificity. XOR is involved in the last 2 steps of purine metabolism; it breaks down hypoxanthine into xanthine and uric acid (Harrison, 2002), while also generating superoxide in XO and NADH in XDH from electron transfer to oxygen and NAD + respectively (Berry and Hare, 2004) (Figure 1B). The enzyme AOX oxidizes aromatic and aliphatic aldehydes into carboxylic acids (Terao et al., 2016).

There is cross talk between Mo and other transitional metals. Mo salts have been reported to affect the absorption of Fe and Cu in the digestive tract which could be as result of competitive inhibition for receptors found in the epithelial lining of the intestines (Vyskočil and Viau, 1999). Cu is also involved in the synthesis of Mo-co by acting via a metal exchange mechanism in the Mo-co precursor molybdopterin where it is bound to molybdopterin dithiolene group prior to exchange for Mo in the synthesis of Mo-co (Mendel and Kruse, 2012). Interestingly, the effects of Cu are dose dependent: a concentration of 1 µM of copper chloride was found to inhibit this metal exchange mechanism (Kuper et al., 2004). It is hypothesized that Mo can react with sulfide to form thiomolybdate which chelates Cu, and the reaction of Cu with this compound reduces its availability to bind to its transporter ceruloplasmin (Vyskočil and Viau, 1999; Dick et al., 1975; Gould and Kendall, 2011).

## 3 Molybdenum: the double-edged sword

**FIGURE 2:**
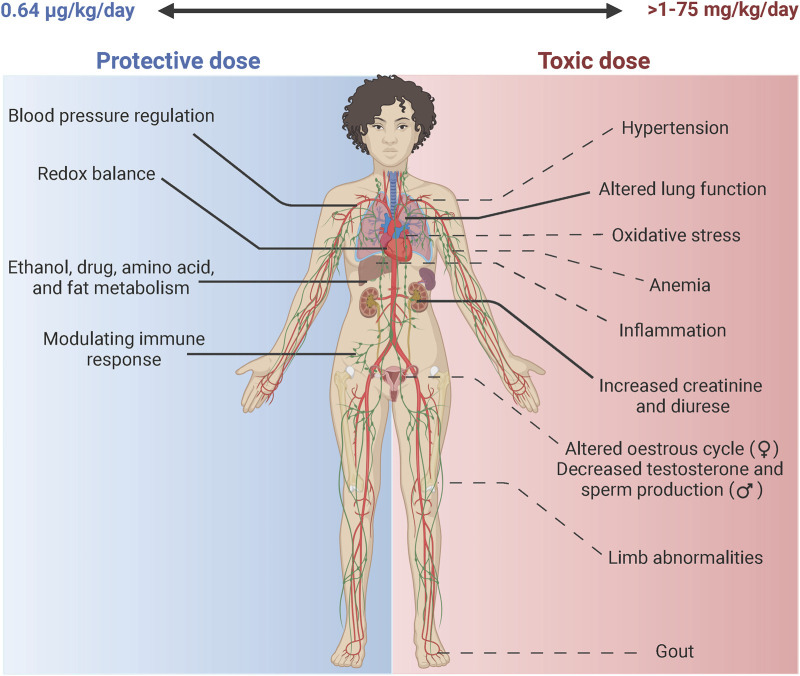
Molybdenum: The double-edged sword. Mo is a bio essential element in the body. The daily nutritional recommendation for Mo is 0.64 ug/kg/day (ATSDR, 2020). At this physiological dose Mo is incorporated in enzymes such as XOR that aids in the maintenance of blood pressure through production of uric acid that activates the renin angiotensin system, Mo also helps maintain redox balance through production of ROS (superoxide& H_2_O_2_) and RNS (NO) species (Bortolotti et al., 2021). Similarly, it facilitates metabolism of drugs, amino acid and ethanol through the activities of AOX (Terao et al., 2016). It acts to modulate the immune response through activation of enzymes XOR in leukocyte cells (Polito et al., 2021). At higher doses Mo can have toxic effects at doses ranging from 1 to 75 mg/kg/day (ATSDR, 2020). According to a systemic review, the highest risk (-- - -) is associated with altered respiratory and renal function extrapolated from human and animal studies (ATSDR, 2020). Other potential toxic effects (-- - -) include effects related to Cu deficiency that include weight loss and anemia, altered reproductive function in both female and male, changes in bone mass specifically in the femur, gout-like symptoms and hypertension due to over production of uric acid. Increased oxidative stress has been reported in *Drosophila melanogaster* (Perkhulyn et al., 2017).

Mo is a trace element (Vyskočil and Viau, 1999) and its bioavailability is critical to metabolic enzymes that are necessary for maintaining redox balance. However, Mo toxicity has also been reported and therefore highly suggests a dose dependent effect in biological environments (Figure 2). At low doses (0.64 μg/kg/day) as a nutritional element (ATSDR, 2020), the vital functions of Mo are achieved as a co factor in enzymes XOR and AOX.

### 3.1 The protective functions of Mo in the body

#### 3.1.1 XOR

XOR is responsible for 3 enzymatic functions: XOR activity that results in uric acid production from hydroxylation of hypoxanthine and xanthine, NADH oxidase activity that produces the reactive oxygen species (ROS) superoxide and consequently hydrogen peroxide (H_2_O_2_), and nitrate reductase activity that produces the reactive nitrite species (RNS) nitric oxide (NO) (Harrison, 2002). XOR is found in the liver, small intestines, mammary glands, cardiac and endothelial cells (Polito et al., 2021). At appropriate levels, these molecules all play a role in maintaining diverse physiological homeostatic functions which include blood pressure regulation, modulating the innate immune response, redox signaling and fat metabolism (Polito et al., 2021). In leukocytes, XO generates superoxide and consequently H_2_O_2_ as the body’s first mechanisms of defense (Polito et al., 2021). Uric acid produced from XOR in adequate amounts can act as an antioxidant by scavenging free radicals. In addition, uric acid regulates blood pressure through the activation of the renin angiotensin system (Battelli et al., 2018).

#### 3.1.2 AOX

AOX is known to oxidize aldehyde and aromatic compounds (Kelley, 2017). It has four isoforms that have been discovered to date, AOX1-AOX4 (Terao et al., 2016). The isoform AOX1 is found in humans and is hypothesized to be involved in the metabolism of amino acids (Isoleucine, valine, tryptophan, tyrosine, and isoleucine) and vitamin metabolism (B6, retinol, nicotinate and nicotinamide) (Terao et al., 2016). AOX1 also plays a role in maintaining the epithelial lining of lung cells (Shintani et al., 2015), adipogenesis and lipid homeostasis (Terao et al., 2016). Another major role of AOX1 is in the metabolism of antitumor drugs, immune modulating, and antiviral agents (Terao et al., 2016). AOX1 also facilitates ethanol metabolism following alcohol consumption (Shaw and Jayatilleke, 1990). Moreover, it has also been shown to produce NO especially under hypoxic conditions (Kelley, 2017).

### 3.2 Toxicity of Mo in the body

The toxicity of Mo is mainly achieved by the molybdate ion (MoO_4_^2-^), in which Mo has an oxidation state of Mo(VI) (Burzlaff et al., 2017). Mo(VI) is the form that is present in blood, up taken by cells, and bound to Mo-Co. The mechanism of Mo toxicity is still under investigation. However, extensive studies have reported a possible association with Cu deficiency, or a condition termed as molybdenosis that mimics Cu deficiency (Barceloux, 1999; ATSDR, 2020). These effects were observed in ruminant models that presented as a decrease in weight, hyperpigmentation, and abnormalities in hair texture (ATSDR, 2020). In human studies, individuals who present with low dietary intake of Cu or impairment of Cu metabolism have also been reported to be at greater risk of Mo toxicity (Vyskočil and Viau, 1999). Some of these cases also showed increased excretion of Cu and increased levels of ceruloplasmin, alluding to a possible antagonistic relationship between Mo and Cu. Supraphysiological levels of Mo via oral ingestion, > 1–75 mg/kg/day has demonstrated toxicity (Vyskočil and Viau, 1999), (ATSDR, 2020). A comprehensive systemic review that assessed both human and animal studies found that the highest presumed risk of Mo toxicity in humans was in the alteration of respiratory and renal function (ATSDR, 2020). Other organ systems affected by high doses of Mo are presented in Figure 2. Below, we briefly summarize the findings on Mo toxicity in the renal and respiratory systems with special emphasis on the cardiovascular system and its implications in ACD.

#### 3.2.1 Nephrotoxicity

In animal studies, histopathological changes in kidney structures have been reported as well as changes in renal function. Oral ingestion of 60 mg/kg/day for a duration of 90 days in female rats resulted in pathological changes in the proximal tubules of the nephron (Murray et al., 2014). Doses of 240 mg/kg/day in another study did cause deleterious effects in kidney histological structure in male rats (Bandyopadhyay et al., 1981). Furthermore, at doses of 80 mg/kg/day, renal function was altered through changes in diuresis and urinary creatinine levels (Bompart et al., 1990) These studies present with convincing evidence of the effects of high dose Mo on renal structure and function. However, its crucial to determine whether these reported effects translate to human studies.

#### 3.2.2 Respiratory toxicity

Airborne exposure of Mo in the form of Mo trioxide (MoO_3_) in human and animal studies has to a certain extent resulted in alterations in lung function. A cohort study demonstrated that workers exposed to MoO_3_ experienced cough, dyspnea, altered lung function tests and radiographic abnormalities (Ott et al., 2004). However the authors did report limitations to the study that included insufficient monitoring and inappropriate control groups. In rats and mice studies, the chronic exposure of Mo at concentrations greater than 6.7 mg Mo/m^3^ and 67 mg Mo/m^3^ respectively, led to extensive histopathological changes in the respiratory and olfactory tracts (Program, 1996).

#### 3.2.3 Cardiovascular toxicity

An association between elevated Mo urinary levels and high blood pressure has been reported in a human cross-sectional study (OR 1.45, 95% CI 1.04–2.02) (Shiue and Hristova, 2014). On the other hand, Schroeder and Kraemer (Schroeder and Kraemer, 1974) found a week negative association between atherosclerotic disease and Mo levels in water consumption (0.00003 mg/kg/day for an assumed daily intake of 2 L per day). These contradicting studies to a certain extent support a dose dependent effect of Mo. As mentioned previously, Mo is an essential element that is incorporated as a co factor in enzymes. One of these enzymes’ XOR plays a significant role in cardiovascular physiology and ACD pathology (Polito et al., 2021). XOR generates ROS (superoxide, H_2_O_2_), and RNS (NO) at the Mo-Co binding site (Bortolotti et al., 2021). At physiological doses these molecules mediate cell signaling that help in maintaining vascular homeostasis (He et al., 2019). For example, NO aids in vessel dilation and inhibiting platelet aggregation while H_2_O_2_ regulates vascular smooth muscle cell proliferation, migration, and differentiation (He et al., 2019; Byon et al., 2016). However, in disease states, there is an overactivation of these enzymes that can cause excessive production of ROS and RNS which can have detrimental effects. XOR has been identified as a factor that contributes to endothelial dysfunction by reducing NO availability (Polito et al., 2021). Superoxide reacts rapidly with NO to produce the toxic RNS species peroxynitrite (Beckman and Koppenol, 1996). It is well documented that ROS and RNS can cause lipid oxidation (He et al., 2019; White et al., 1994; Leeuwenburgh et al., 1997). In fact, Ox-LDL is one of the main factors in the underlying mechanism of ACD progression. Moreover, XOR has been identified within atherosclerotic plaques isolated from tissue obtained from carotid endarterectomy (Patetsios et al., 2001). Macrophages found within atherosclerotic plaques in mice have also been found to overexpress XOR (Nomura et al., 2014). Uric acid at high levels is also associated with endothelial dysfunction, proliferation of smooth muscle cells, and activation of the renin-angiotensine system (Polito et al., 2021). Therefore, it is not surprising that Mo levels have been found to be associated with hypertension and atherosclerotic disease. In fact, individuals residing in areas with high Mo intake not only had high serum Mo levels, but this was also in association with increased XO and uric acid levels (Kovalskiy et al., 1961). A recent animal study showed that Mo increases the risk of ACD progression in Apo E^−/−^ mice exposed to Mo disulfide nanosheets (MoS_2_ NSs) (Yan et al., 2023). Taken together, this work suggests that Mo levels at the degradation site could potentially influence ACD pathophysiology and this effect could be dose dependent.

## 4 Biomedical research studies of molybdenum for cardiovascular applications

In addition to Mo’s biological significance, metallic Mo presents with excellent mechanical properties that surpasses all biodegradable metals considered to date for ACD (Table 1). These include a high tensile strength of 1,400 MPa, sufficient stiffness (Young’s modulus) estimated at 324 GPa while also maintaining a high elongation to failure of up to 50% (Redlich et al., 2021). Mo is also degradable with a uniform, slow and steady degradation rate (Redlich et al., 2021; Schauer et al., 2021). Moreover, due to its radio-opacity it can have applications in medical imaging (Schauer et al., 2021; Yan et al., 2023) Collectively, these characteristics make it the ideal material to use for biomedical vascular implants which would provide a temporary scaffold while facilitating tissue repair over time. Therefore, it is not surprising that it is currently being considered as a treatment alternative to permanent stents for ACD (Schauer et al., 2021; Sikora-Jasinska et al., 2022; Bian et al., 2024). Below, we will discuss the *in vitro* and *in vivo* work that has been done to assess the biocompatibility of Mo with a focus on cardiovascular applications. We also present studies performed on invertebrate models that allude to the Mo effect as a regulator of mechanisms that directly or indirectly play an active role in ACD.

**TABLE 1:** The comparison of biocompatibility properties of biodegradable metallic materials.

Material Biocompatibility Property	Iron (Fe)	Zinc (Zn)	Magnesium (Mg)	Molybdenum (Mo)
Physiological response	Essential element important for oxygen transport (Lieu et al., 2001; Vogt et al., 2021)	Essential trace element that affects enzyme functionality (Vallee, 1988), (Coleman, 1998), (Chasapis et al, 2020)	Essential for biochemical processes, bone, and muscle formation (Standing Committee on the Scientific Evaluation of Dietary Reference Intakes, 1999; Fiorentini et al., 2021)	Essential trace element, cofactor for metabolic enzymes (Schwarz et al., 2009; Maia et al., 2024)
Cell viability	>80% viability after 48 h with 99.95% extract *in vitro* using Adipose-derived stem cells (ADSCs) (Paim et al., 2020)	<80% viability with pure extract *in vitro* using SMC and Endothelial cells (Fu et al., 2020)	≥80% viability with pure extract *in vitro* using SMC and Endothelial cells (Fu et al., 2020)	Signs of cell death only occurred with 2.5 mM after 48 h (Redlich et al., 2021)
Inflammatory response	No significant inflammatory response *in vivo* (Li et al., 2014; Xu et al., 2023)	Moderate inflammatory response, possibly due to slower *in vivo* degradation (Fu et al., 2020; Oliver et al., 2020; Guillory et al., 2022)	Generally low inflammatory response which decreased after *in vivo* degradation (Fu et al., 2020)	Limited studies suggest no significant inflammatory response (Schauer et al., 2021)
Mechanical properties: ultimate tensile strength (MPa)	High, varies with alloying: ∼290 (Moravej et al., 2010; Salama et al., 2022)	Moderate, varies with alloying: ∼120 (Bowen et al., 2016; Cockerill et al., 2021)	Low, varies with alloying: ∼86 (Agarwal et al., 2016; Song et al., 2020)	High, ∼1,400 (Schauer et al., 2021)
Young’s modulus (GPa)	∼200 (Song et al., 2014), (Hermawan, 2018)	∼100 (Hermawan, 2018), (Ledbetter, 1977), (Shi et al., 2020)	∼45 (Hermawan, 2018)	∼320 (Schauer et al., 2021)
Ductility	High (Li et al., 2014; Korei et al., 2022)	Moderate (Fu et al., 2020)	Low, varies with alloying (Li et al., 2014; Song et al., 2020)	High (Sikora-Jasinska et al., 2022)
Elongation to failure	Up to 40% (Rybalchenko et al., 2022)	Varies with alloying (Guillory et al., 2022)	Varies with alloying (Song et al., 2020)	Up to 50% (Schauer et al., 2021)
Degradation properties	Uniform (Li et al., 2014)	Uniform (Bowen et al., 2013b; García-Mintegui et al., 2021)	Non uniform (Lee et al., 2009)	Uniform degradation (Sikora-Jasinska et al., 2022; Schauer et al., 2021)
Estimated degradation Rate	100 μm/y *in vitro* (Hermawan, 2018)	160 μm/y *in vitro* (Hermawan, 2018)	4,000–8,000 μm/y *in vitro* (Hermawan, 2018)	33.6 μm/y *in vitro*, 13.5 μm/y *in vivo* (Schauer et al., 2021)

### 4.1 *In vitro* models

Redlich et al. (2021) published the first extensive study that assessed the degradation behavior and effect of metallic Mo on vascular and immune cells actively involved in the inflammatory response of ACD. The degradation rate in simulated biological fluids after immersion of Mo for 28 days was calculated to be approximately 10.1 ± 0.7 and 10.8 ± 0.6 μm/year in two different preparations of Mo (commercially available and powdered metallurgically produced Mo respectively). A subsequent study reported a degradation rate of 33.6 μm/year for the same duration in a modified solution with decreased calcium content (Schauer et al., 2021). These results are in comparison to an ideal degradation rate of 20 μm/y for biodegradable stent materials (Bowen et al., 2013a; Schauer et al., 2021). Cells were cultured in growth media that contained varying concentrations of MoO_3_, which is the end dissolution product of metallic Mo. This compound ionizes in solution to form MoO_4_^2-^. The experiments were run for 24–72 h. Endothelial cells cultured at 2.5 mM MoO_3_ showed a decrease in cell viability from 48 h compared to the control group. Human coronary artery smooth muscle cells showed greater resiliency by maintaining appropriate cell viability in all concentrations used. Moreover, exposure of human thrombocytes to Mo wires did not trigger the expression of thrombogenic markers. In human monocytes, only 2.5 mM upregulated the gene expression of the inflammatory cytokines IL-1, IL-8 and TNF-α at 24 h of treatment. In addition, these cells were able to colonize and migrate over Mo materials proving Mo ability to provide a suitable scaffold that facilitates vascular remodeling and repair.

Prieto Jarabo et al. (2024) assessed biocompatibility of Mo for applications in temporary cardiac pacing that is often performed to treat cardiac arrhythmias that manifest after cardiac surgery. The authors showed that Mo consistently maintained a uniform degradation profile when incubated in simulated body fluids for at least 28 days. Low cytotoxicity was observed in human cardiomyocytes and cardiac fibroblasts. Although, the Mo effect was both time and dose dependent in some instances. For example, apoptosis was increased at low concentrations of Mo at 24 h in cardiomyocytes while necrosis decreased in cardiomyocytes at the same time period for the highest Mo concentrations.

An *in vitro* study which investigated the effect of Mo nanoparticles on human epidermal epithelial cells showed that exposure of up to 1 mg/mL of MoO_3_ did not result in significant cell death (Božinović et al., 2020). However, the initiation of signaling cascades that led to upregulated levels of Il-6 and excessive cell growth were observed. Upregulation of inflammatory cytokines as well as vascular smooth muscle cell hyperproliferation is seen in the ACD progression (Rafieian-Kopaei et al., 2014). Therefore, these mechanisms could also have implications in Mo cardiovascular implants.

The treatment with doses of MoS_2_ of up to 50 μg mL^-1^ on M1 and M2 macrophages showed that although there was no significant impact on viability, M1 showed increased secretion of inflammatory cytokines (Lin et al., 2020).

An Mo stent was recently engineered from Mo powder using selective laser melting (SLMed Mo) (Bian et al., 2024). The biological response of the stent was assessed using biodegradation products, cell colonization on the stent, cell apoptosis and hemocompatibility. Overall, SLMed Mo showed superior cell viability, reduced apoptosis, adequate colonization of endothelial cells and vascular smooth muscle cells compared to Zn and Mg. Moreover, SLMed Mo showed minimal signs of hemolysis and coagulation.

Currently, only one study has investigated the effects of Mo under simulated inflammatory conditions which would better represent the biological environments that vascular stents would be exposed to (Shen et al., 2024). The authors incubated Mo with H_2_O_2_ and Fenton reagent which produces the highly reactive species hydroxyl radical (HO.) (Shen et al., 2024). Mo maintained uniform degradation in the inflammatory and control conditions (PBS). However, the authors did report an increase in the degradation rate of Mo with increasing concentration of the inflammatory species. The products of degradation in simulated inflammation were predominantly MoO_2_ and MoO_3_. MoO_3_ dissolves to form MoO_4_^−2^ which is the predominant Mo compound formed even under physiological conditions.

### 4.2 *In vivo* invertebrate models

Invertebrate models such as *Drosophila Melanogaster* (*Drosophila*) have gained popularity in research for studying molecular mechanisms of various biochemical molecules. For Mo, some of these models demonstrate a similar response to humans when exposed to Mo. For example, investigative studies of Mo in *Drosophila* have been shown to upregulate AOX and XDH enzymatic activity following Mo treatment (Duke et al., 1975). *Drosophila* treated with sodium molybdate (Na_2_MoO4) at different concentrations ranging from 0.025-10 mM showed a dose dependent effect on oxidative stress and antioxidant markers (Perkhulyn et al., 2017). Low concentrations of MO enhanced antioxidant capacity and mild oxidative stress, while Mo at high concentrations induced intermediate or high intensity oxidative stress. This study also reported sex dependence. For example, oxidative stress molecules such as protein carbonyl content was lowered by 24% at 10 mM in males while it increased by 33% at the same concentration in females. In addition, lipid peroxide concentrations increased in both males and females. The authors also showed that the antioxidant superoxide dismutase increased by 58% with 10 mM Mo treatment for males, while it showed no significant difference in the female for the same treatment group.

Mo has also been found to decrease blood glucose levels by improving glucose utilization within cells in mammalian studies (Rovenko et al., 2014) This effect was also reproduced in *Drosophila* treated with 0.025–10 mM concentrations of Na_2_MoO4. Hemolymph glucose levels were found to be decreased significantly in males (between 21%-42% for all concentrations), while females had varying results depending on strain. Glycogen levels were increased in both males and females. While important, investigating these dose dependent effects of Mo on antioxidant and oxidative stress markers of ACD in a mammalian model would be an important next step to further validate these findings or determine how the model organism impacts the effects of Mo.

### 4.3 *In vivo* mammalian models

Similar to *in vitro* studies, Mo has been shown to exhibit uniform degradation properties, with a reported degradation rate of 13.5 μm/y (Schauer et al., 2021). The implantation of pure Mo into the abdominal aorta of rats for periods ranging from 3-12 months showed no significant changes in urine or blood levels of Mo. There were undetected C-reactive protein levels, which is a clinical indicator of systemic inflammation. The authors also observed a decrease in the blood cell count which returned to baseline over time. There were no adverse effects or morphological changes in the kidney or liver reported. Although, high Mo concentrations were detected in close vicinity to the implant site (Schauer et al., 2021).

The biocompatibility of Mo was further supported in a subsequent study which showed low levels of inflammation and comparable neointimal thickness when compared to the platinum control after 6 months implantation in the abdominal aorta (Sikora-Jasinska et al., 2022). However, there was a non-significant decrease in reendothelialization of the abdominal aorta in Mo implanted mice, and some areas of the kidney showed abnormal structural changes, which could possibly indicate some level of undesirable vascular and renal effects.

Bian et al. (2024) implanted the SLMed Mo stent adjacent to the abdominal aorta of a rat model to validate Mo biocompatibility. After 3 months of implantation, there were no signs of necrosis, cell hyperproliferation or aggressive inflammation. The results are encouraging. Although, the experimental design does not clearly depict the expected biological response since a stent is implanted within the intima of the vessel wall.

## 5 Toxicity of Mo compared to other biodegradable metal elements

All the biodegradable metals considered for vascular stents are bio essential elements that are needed to regulate homeostasis (Table 1). However, at supraphysiological doses, they exhibit toxicity at the implant site and in different organ systems, which is one of the concerns for their application. Zn presents with toxic effects at exposures >1 g. The exact mechanism of Zn toxicity is not clearly understood, short term exposure can manifest as digestive and respiratory system complications as well as immune-mediated inflammation. Long term exposure can affect the nervous system as well as disruption within the hematological system, specifically the bone marrow (Agnew and Slesinger, 2020). Zn has been found to have dose dependent effect on caspase 3 mediated apoptosis in vascular smooth muscle cells (Guillory et al., 2020). In fact, it was shown to suppress neo intima formation in the blood vessel wall (Bowen et al., 2015; Guillory et al., 2020).

Fe toxicity occurs at consumption of 20–60 mg/kg for mild to life-threatening cases respectively, typically from Fe supplements (Yuen and Becker, 2023). Excess Fe leads to its accumulation in the mitochondria of cells where it interferes with oxidative processes, causing an increase in free radicals and ultimately cell and tissue damage (Kumar Baranwal and Singhi, 2003). This usually affects the metabolic activity of cells mainly in the cardiovascular, nervous, and digestive systems. At the implant site, Fe based materials produce ROS species such as H_2_O_2_ and HO. which could lead to chronic inflammation and damage to the surrounding tissue (Scarcello and Lison, 2020). Although *in vivo* studies performed to date report minimal inflammatory effects (Scarcello and Lison, 2020). In fact, a recent clinical study reported the safety and competency of Fe based stents in patients with non-complicated coronary lesions (Gao et al., 2023).

A substantial amount of Mg (5,000 mg/day) is needed to reach toxic levels, and this presents with diarrhea and vomiting, arrythmias, loss of muscular strength, decreased urine output and impairment of the central nervous system in severe cases (Musso, 2009). Mg has been reported to have dose-dependent effect on cell viability as well as increased red blood cell lysis which is most likely due to pH changes during degradation rather than increased Mg ion concentration (Zhen et al., 2015). Mg based stents have been extensively applied in human studies and one of the reasons is associated with its low toxicity.

Compared to these metals, Mo presents with the lowest dose limit of toxicity (1–25 mg/kg/day) in animal studies following oral administration (ATSDR, 2020). However, the current research studies outlined in this review for its application as a vascular stent have not demonstrated many of the toxic effects associated with Mo (Figure 2).

## 6 Limitations of current research

This review set out to explore the biocompatibility of Mo as a biomaterial for vascular stent application by first understanding the biochemical and physiological aspects of Mo which plays a crucial role in the function of metabolic enzymes. Specifically, XOR is at the frontline in the pathogenesis of ACD (Polito et al., 2021). The research studies *in vitro* and *in vivo* provide compelling evidence that supports the use of Mo as cardiovascular implant. However, the underlying mechanisms involved in the functionality of XOR that could potentially drive Mo toxicity at the implant site have not been explored. XOR has been identified in macrophage and endothelial cells (Polito et al., 2021; Berry and Hare, 2004). These are the cells that come into initial contact with the implant. Moreover, the ability for endothelial cells to migrate over the stent is an attractive quality for vascular implants because the endothelial lining reduces the risk of thrombogenicity and neointima hyperplasia (Jukema et al., 2012; He et al., 2019). Endothelial cells in both *in vivo* and *in vitro* experiments appear to be susceptible to damage from Mo degradation products at high doses (Redlich et al., 2021; Sikora-Jasinska et al., 2022). In addition, the consideration of the inflammatory response in both physiological and pathological conditions is vital to the success of these implants and only one study to date has investigated the effect of inflammatory species on degrading Mo (Shen et al., 2024). Mg based materials demonstrated adequate biocompatible properties in preclinical studies. Oliver et al. (2021) However, the translation to clinical application has been disappointing especially in individuals with underlying comorbidities, which would be indicative of chronic inflammatory states (Yang et al., 2018; Peng et al., 2019). Moreover, recent *in vitro* studies showed that Mg materials elicit significantly different host and material interactions in quiescent compared to inflammatory macrophage environments (Kwesiga et al., 2023). The cross talk of Mo and Cu needs to be critically assessed in biomedical research studies since it is well described that there is a counteractive functional relationship that exists between Mo and Cu, which could have molecular and clinical implications especially in individuals who are susceptible to Cu deficiency. Another drawback is that most studies on Mo toxicity have looked at its oral or airborne intake. This makes it challenging to extrapolate the results to Mo distribution within the human circulatory system. There are also ethical and cost concerns that limit extensive investigation into the biological implications of Mo based biomaterials in mammalian species. Further research with feasible experimental models that account for the intricate and multifaceted biological role of Mo in ACD pathogenesis are still needed.

## 7 Future outlook

**FIGURE 3:**
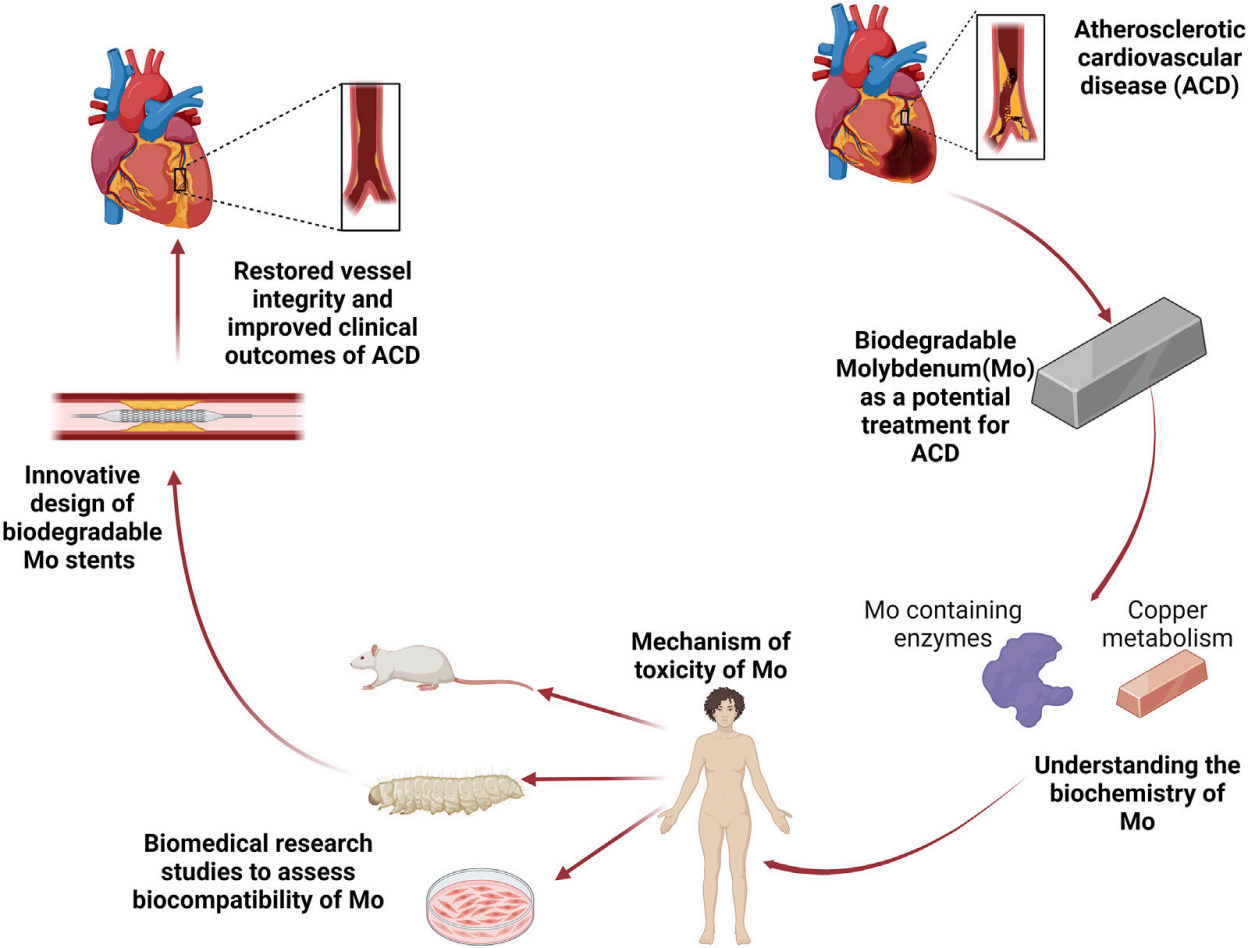
The overall perspective. Atherosclerotic cardiovascular disease (ACD) is the leading cause of death worldwide. Biodegradable Molybdenum (Mo) has the potential to address the complications of current treatments for ACD. A better understanding of Mo biochemistry that includes its incorporation in metabolically active enzymes and cross talk with Cu will facilitate the possible mechanisms of toxicity which can guide biomedical research studies, improve stent design, and consequently restore vascular function.

Mo requiring enzymes play a critical role in maintaining nitroso redox balance within cells (Kelley, 2017). This balance is altered in ACD and might have implications in the degradation properties of Mo. The generation of ROS and RNS from macrophages has been shown to modulate the degradation of Mg materials (Kwesiga et al., 2023). In fact, as previously mentioned, Mo under simulated inflammatory conditions show changes in the degradation rate when incubated in ROS (Shen et al., 2024). Furthermore, the function of XOR is altered in ACD which leads to over production of ROS and toxic RNS, ultimately leading to oxidative stress which is driver of ACD pathogenesis (Polito et al., 2021). To our knowledge, it remains unknown on how the degradation of Mo would alter XOR activity especially under inflammatory environments. *In vitro* work on Mo and cardiovascular functionality should not only explore cell viability and inflammatory cytokines but also determine whether Mo degradation products affects XOR activity in cells such as macrophages and endothelial cells. Additional studies investigating the effects of Mo in a minimally invasive and cost efficient *in vivo* model are of interest to determine the safety of Mo containing biodegradable metals for stent applications at the biochemical and molecular levels. *Galleria mellonella (galleria)* is a recently upcoming invertebrate model organism for immune and toxicological studies (Coates et al., 2019; Wojda et al., 2020), which offers a method to facilitate this investigation. One of the main advantages of galleria is that its innate immune system shares similarities with humans (Wojda et al., 2020). However traditional methods for obtaining biochemical information, such as western blotting, flow cytometry, or gene sequencing, are cost inefficient and do not maintain crucial spatial data. Autofluorescence imaging of insect models is a promising alternative, biological systems are rich in endogenous fluorophores that can be used for autofluorescence molecular imaging in a convenient, label-free manner (Quansah et al., 2022). Endogenous fluorophores are powerful biomarkers because their emission properties are often influenced by their microenvironment, as well as the morphology, metabolic state, and pathological conditions of the sample. This technique has been extensively used in human and animal models to study lipids, NAD(P)H, and collagen, all of which are well-known molecular markers for describing inflammation (Kretschmer et al., 2016; Chernavskaia et al., 2016). The translation of *in vitro* work to determine the alterations in the degradation profile of Mo and host response in an invertebrate model would greatly improve the ability of research scientists and engineers to accurately predict the performance of Mo implants. Lastly, the cross talk between Mo and Cu remains an intriguing area of study, and it would be of interest to determine if the antagonistic relationship would affect Cu metabolism after implantation, and whether the Mo/Cu interaction could alter the degradation dynamics of Mo at the implant site. Figure 3 presents the overall perspective of Mo towards its application for the treatment of ACD.

## 8 Conclusion

Atherosclerotic cardiovascular disease remains the leading cause of death and morbidity worldwide. Although the deployment of permanent stents has been a lifesaving treatment. There are still complications that can potentially be addressed by biodegradable metal stents. Molybdenum, a metallic bio essential element that presents with excellent mechanical properties can provide the temporary scaffold that the blood vessel needs to heal and eventually degrade after vascular function is restored. The toxicity of Mo has been explored in various organ systems and the biocompatibility studies of Mo for cardiovascular applications shows minimal toxicity. However, further studies that emphasize on molecular mechanisms directly involved in the biological role of Mo in physiological and pathological environments could greatly improve our understanding of the Mo and host interaction, direct material design and ultimately the success of Mo as a biodegradable vascular stent.

## Abbreviations

ACD, Atherosclerotic cardiovascular disease; AOX, Aldehyde oxidase; Cu, Copper; Mo, Molybdenum; FAD, Flavine adenine dinucleotide; Fe, Iron; FeMo-co, Iron–sulphur-cluster-based iron–Mo cofactor; H_2_O_2_, Hydrogen peroxide; HO, Hydroxyl radical; Mo, Molybdopterin co factor; MoO_3_, Mo trioxide; MoO_4_^2-^, Molybdate ion; MOT Molybdenum transporter; Ox-LDL, Oxidized low density lipoprotein; NO, Nitric oxide; Na_2_MoO_4_, Sodium molybdate; NAD (P) H, Nicotinamide adenine dinucleotide phosphate/Nicotinamide adenine dinucleotide; ROS Reactive oxygen species; RNS, Reactive nitrogen species; NAD, Nicotinamide adenine dinucleotide; XO, xanthine oxidase; XOR, xanthine oxidoreductase; XDH, Xanthine dehydrogenase; Zn, Zinc.
